# FGF18, a prominent player in FGF signaling, promotes gastric tumorigenesis through autocrine manner and is negatively regulated by miR-590-5p

**DOI:** 10.1038/s41388-018-0430-x

**Published:** 2018-08-06

**Authors:** Jinglin Zhang, Yuhang Zhou, Tingting Huang, Feng Wu, Yi Pan, Yujuan Dong, Yan Wang, Aden K. Y. Chan, Liping Liu, Johnny S. H. Kwan, Alvin H. K. Cheung, Chi Chun Wong, Angela K. F. Lo, Alfred S. L. Cheng, Jun Yu, Kwok Wai Lo, Wei Kang, Ka Fai To

**Affiliations:** 1 Department of Anatomical and Cellular Pathology, State Key Laboratory of Oncology in South China, Prince of Wales Hospital, The Chinese University of Hong Kong, Hong Kong SAR, People’s Republic of China; 2 Institute of Digestive Disease, Partner State Key Laboratory of Digestive Disease, The Chinese University of Hong Kong, Hong Kong SAR, People’s Republic of China; 3 Li Ka Shing Institute of Health Science, Sir Y.K. Pao Cancer Center, The Chinese University of Hong Kong, Hong Kong SAR, People’s Republic of China; 4 Shenzhen Research Institute, The Chinese University of Hong Kong, Shenzhen, People’s Republic of China; 5 Key Laboratory of Cardiovascular Medicine and Clinical Pharmacology of Shanxi Province, Shanxi Medical University, Taiyuan, People’s Republic of China; 6 Department of Hepatobiliary and Pancreatic Surgery, Shenzhen People’s Hospital, Second Clinical Medical College of Jinan University, Shenzhen, Guangdong Province People’s Republic of China; 7 School of Biomedical Sciences, The Chinese University of Hong Kong, Hong Kong SAR, People’s Republic of China; 8 Department of Medicine and Therapeutics, The Chinese University of Hong Kong, Hong Kong SAR, People’s Republic of China

**Keywords:** Gastric cancer, Oncogenes

## Abstract

Fibroblast growth factors (FGFs) and their receptors are significant components during fundamental cellular processes. FGF18 plays a distinctive role in modulating the activity of both tumor cells and tumor microenvironment. This study aims to comprehensively investigate the expression and functional role of FGF18 in gastric cancer (GC) and elucidate its regulatory mechanisms. The upregulation of FGF18 was detected in seven out of eleven (63.6%) GC cell lines. In primary GC samples, FGF18 was overexpressed in genomically stable and chromosomal instability subtypes of GC and its overexpression was associated with poor survival. Knocking down FGF18 inhibited tumor formation abilities, induced G1 phase cell cycle arrest and enhanced anti-cancer drug sensitivity. Expression microarray profiling revealed that silencing of FGF18 activated ATM pathway but quenched TGF-β pathway. The key factors that altered in the related signaling were validated by western blot and immunofluorescence. Meanwhile, treating GC cells with human recombinant FGF18 or FGF18-conditioned medium accelerated tumor growth through activation of ERK-MAPK signaling. FGF18 was further confirmed to be a direct target of tumor suppressor, miR-590-5p. Their expressions showed a negative correlation in primary GC samples and more importantly, re-overexpression of FGF18 partly abolished the tumor-suppressive effect of miR-590-5p. Our study not only identified that FGF18 serves as a novel prognostic marker and a therapeutic target in GC but also enriched the knowledge of FGF-FGFR signaling during gastric tumorigenesis.

## Introduction

Gastric cancer (GC) is among the severe health problems worldwide [1]. Late-emerging symptoms, increasing metastasis and chemoresistance are the major hindrances for revealing its pathological mechanisms and developing treatment strategies. GC cases are mostly adenocarcinomas, with considerable histological and etiological heterogeneity. Genetic and environmental factors contribute to GC initiation and progression [2]. According to classical Lauren’s criteria, GC is generally subgrouped as intestinal type and diffuse type. With growing genomic discoveries, a new classification was proposed based on large-scale GC cohorts, in which, GC was divided into: microsatellite instability (MSI), Epstein-Barr virus (EBV)-associated GC (EBVaGC), chromosomal instability (CIN), and genomically stable (GS). Genetic features of each molecular subtype are distinct. For instance, MSI exhibits more frequent incidence of somatic mutations while EBVaGC has the propensity for genome-wide hypermethylation [3]. Novel findings of genetic features are conducive to uncover the molecular mechanisms and provide effective therapeutic targets for GC.

Fibroblast growth factors (FGFs) comprising 22 secretion proteins, which were divided into seven subfamilies. FGF receptors (FGFRs) consist of four homologs (FGFR1 to 4). They are highly conserved transmembrane tyrosine kinase receptors (TKRs) [4]. The FGF-FGFR cascade is a multifactorial intracellular pathway that contributes to a broad range of biological events, such as tissue development, angiogenesis, and tissue regeneration [5]. FGF-FGFR signaling pathways have been implicated in the development a variety of tumors, whose activation increases the motility and invasiveness of cancer cells [6]. In a few studies, particularly, the involvement of FGF18 has been identified under the context of cancer development [7–10]. In colorectal cancers for instance, FGF18 is upregulated through the constitutive activation of the Wnt signaling, suggesting the role of FGF18 as a downstream target of β-catenin [11]. Although the clinical relevance of FGF18 has been described in cancers, its underlying pathophysiological role in tumor progression remains elusive.

Thus in this study, we will comprehensively reveal the expression and clinical correlation of FGF18 in GC, and perform a deep investigation on how FGF18 is activated and promotes gastric carcinogenesis. We aim to identify novel prognostic biomarkers and therapeutic targets for clinical intervention.

## Results

### FGF18 is upregulated in GC and correlates with poor survival

**Fig. 1:**
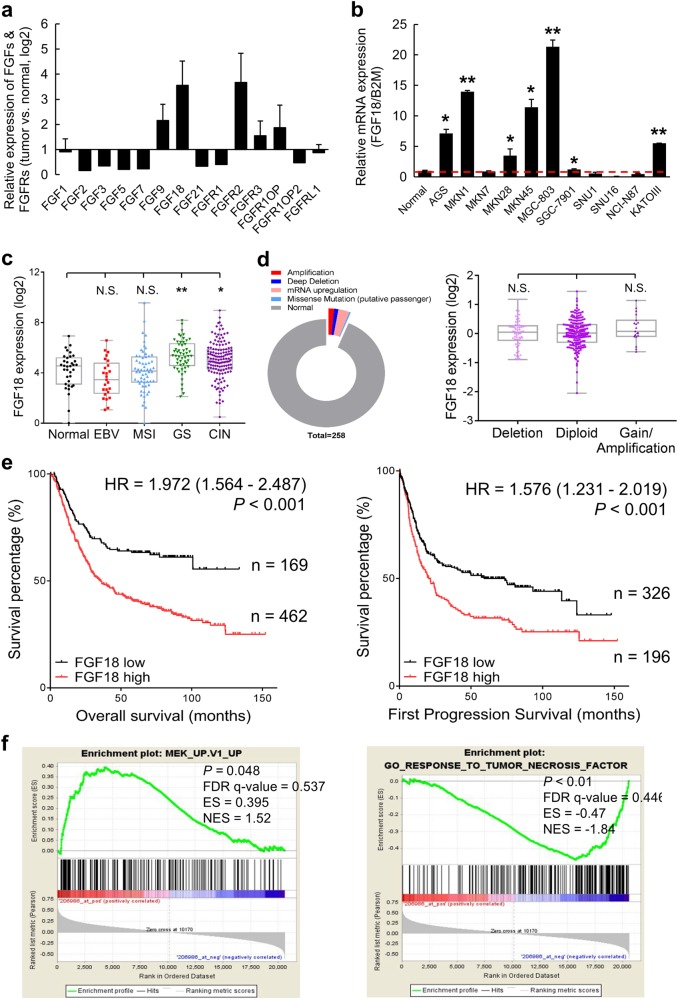
FGF18 shows overabundance in GC. **a** FGF18 has the highest expression level in FGFs and FGFRs among GC cell lines. **b** FGF18 is overexpressed in seven out of eleven GC cell lines (*, *P* < 0.05; **, *P* < 0.001). Dash line indicated the normalized expression of FGF18 in GES-1. **c** Expression pattern of FGF18 based on molecular classification. EBV EBV-positive, MSI microsatellite unstable, GS genomically stable, CIN chromosomal instability (N.S. not significant; *, *P* < 0.05; **, *P* < 0.001). **d** Left panel: Different types of FGF18 genetic alterations in primary GC samples (*n* = 258). Right panel: the correlation of FGF18 mRNA expression with its copy number aberrations (N.S. not significant). **e** Upregulation of FGF18 indicated worse outcomes (*P* < 0.001) based on the Kaplan Meier plotter (www.kmplot.com) analysis. **f** Enrichment plots of gene expression signatures according to FGF18 expression levels in a breast cancer cohort (NCBI/GEO/GSE57303; left panel: MEK signaling, *P* = 0.048; right panel: tumor necrosis factor signaling, *P* *<* 0.01). The barcode plot indicated the position of the genes in each gene set; red and blue colors represented the high and low expression of FGF18, respectively

To detect the expression patterns of FGFs and FGFRs in GC, gene expression microarray profiling was applied to comprehensively reveal the mRNA levels of FGF and FGFR members from 10 GC cell lines, while an immortalized gastric epithelium cell line (GES-1) was used as a control. The mRNA level of FGF18 and FGFR2 were higher than that of other family members in GC cell lines (Fig. 1a). Detecting by qRT-PCR, the relative mRNA expression of FGF18 was upregulated in seven out of eleven (63.6%) GC cell lines (AGS, MKN1, MKN28, MKN45, MGC-803, SGC-7901, and KATOIII; *, *P* < 0.05; **, *P* < 0.001) (Fig. 1b). By analyzing The Cancer Genome Atlas (TCGA) data, FGF18 was found overexpressed in the GS and CIN molecular subtypes (Fig. 1c). Intriguingly, even though *FGF18* harbored deletion or amplification genetically (left panel, Fig. 1d), its copy number gain failed to positively correlate with its abundant mRNA expression (right panel, Fig. 1d), suggesting that translational or post-transcriptional regulation might be responsible for its mRNA upregulation. Moreover, the relation between FGF18 and the survival rate of GC patients was determined by employing Kaplan Meier plotter (www.kmplot.com) in this study. The abundance of FGF18 predicted poor prognosis for GC patients (Fig. 1e). In terms of the mechanism of FGF18 in carcinogenesis, gene set enrichment analysis (GESA) [12, 13] revealed that FGF18 was positively associated with MEK signaling, but negatively correlated with tumor necrosis factor (TNF) signaling (Fig. 1f).

### Silencing FGF18 inhibits tumor growth and promotes cell cycle arrest

**Fig. 2:**
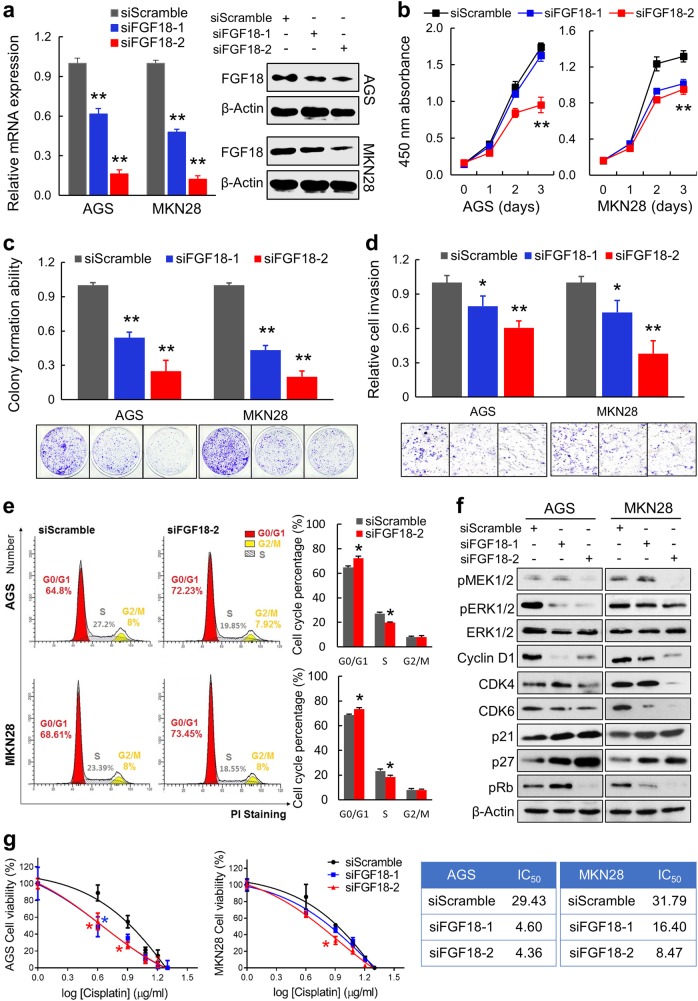
Silencing of FGF18 in GC cells displays anti-tumor function in vitro. **a** Transfection of siFGF18s significantly reduced both the mRNA and protein levels of FGF18 (**, *P* < 0.001). **b** Three-day cell proliferation assays presented that siFGF18-transfection significantly suppressed proliferation in GC cells (**, *P* < 0.001). The mean and SDs obtained from six wells were plotted. **c** Monolayer colony formation assays suggested that siFGF18-transfection inhibited anchorage-dependent colony formation ability (**, *P* < 0.001). Assays were performed in triplicate. Error bars represented SDs. **d** siFGF18-transfected cells showed retarded cell invasion (*, *P* < 0.05; **, *P* < 0.001). Vision fields were randomly picked for thrice, from which the SDs were achieved. **e** Cell cycle distribution examined by flow cytometry indicated G1 arrest in siFGF18-transfected cells. Experiments were performed in triplicate. Statistical analysis of cell cycle percentages was presented by histograms (*, *P* < 0.05). **f** Western blot analysis demonstrated the protein levels of cell cycle regulators, as well as the phosphorylated MEK1/2, ERK1/2 after FGF18 silencing. **g** Drug sensitivity was enhanced by treating cells with siFGF18s (*, *P* < 0.05). The cell viability upon different concentrations of Cisplatin was detected after 48 h by CCK8 cell proliferation assay. The mean and SDs were obtained from 6 wells. The largest mean was defined as 100% and the smallest mean was defined as 0%. IC_50_ values were calculated and listed in the right panel

As FGF18 is upregulated in GC, siRNA-mediated knockdown was used to investigate the functional roles of FGF18 in gastric carcinogenesis. Both mRNA and protein levels of FGF18 were effectively reduced after siFGF18 transfection (Fig. 2a). Accordingly, the proliferative ability, which was revealed by cell proliferation and monolayer colony formation assays, was significantly suppressed by siFGF18 transfection (*P* *<* 0.001, Fig. 2b, c). Meanwhile, cell invasion ability was also inhibited by FGF18 knockdown (*P* < 0.001, Fig. 2d).

Given that cell proliferation was inhibited by FGF18 downregulation, the cell cycle distribution was examined by flow cytometry analysis. After transfected with siFGF18 in both cell lines, G1 phase cells showed a significant enrichment (AGS: 64.8 ± 1.06% vs. 72.23 ± 1.6%, *P* *<* 0.05; MKN28: 68.61 ± 0.75% vs. 73.45 ± 1.22%, *P* *<* 0.05), but S phase cells were reduced (AGS: 27.2 ± 1.11% vs. 19.85 ± 0.5%, *P* *<* 0.05; MKN28: 23.39 ± 1.63% vs. 18.55 ± 1.31%, *P* < 0.05) (Fig. 2e). Cell cycle parameters and regulators were then detected by western blot. Specifically, Cyclin D1, CDK4, CDK6, and pRb were downregulated, while p21 and p27 were activated. Meanwhile, phosphorylated MEK1/2 and ERK1/2 were decreased, indicating low activity of ERK signaling in FGF18-depleted cells (Fig. 2f). To identify the effect of FGF18 on anti-cancer drug treatment, drug sensitivity was assessed. GC cells with or without FGF18-depletion were treated with different concentrations of Cisplatin. Clearly, cells with siFGF18 were more sensitive to this kind of anti-tumor drug (*, *P* *<* 0.05) (Fig. 2g).

### Silencing FGF18 activates ATM but suppresses TGF-β signaling pathway

**Fig. 3:**
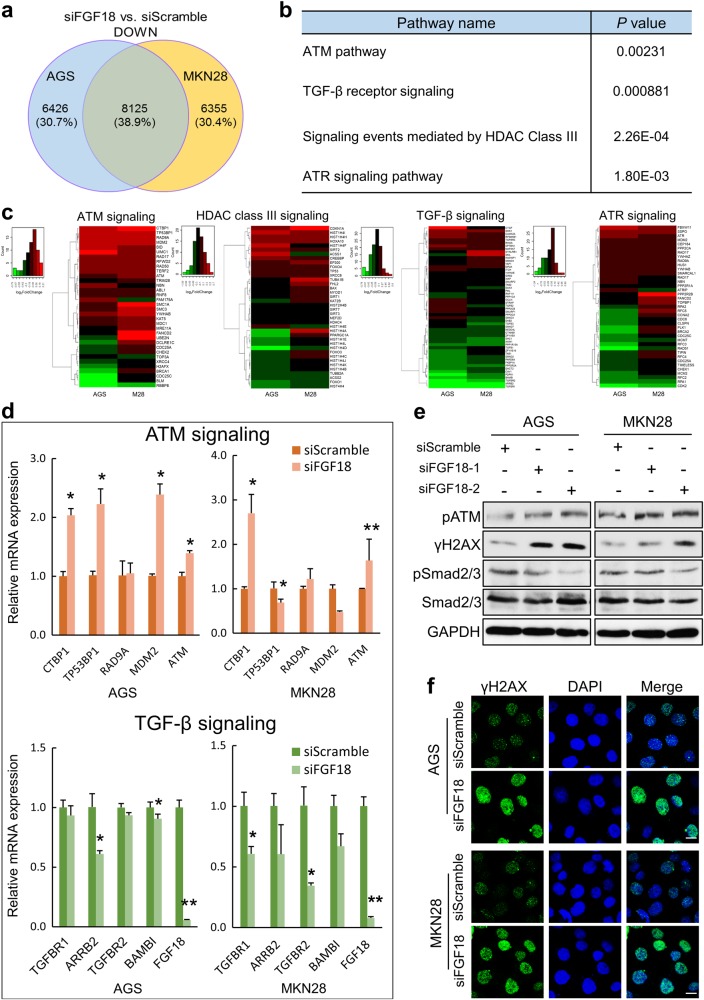
FGF18 crosstalks with ATM and TGF-β pathways. **a** Selection of downregulated genes in both siFGF18-treated cell lines. **b** The genes downregulated in both cell lines with FGF18 knockdown significantly enriched in four signaling pathways. **c** The heat maps demonstrated the differentially expressed genes in these four signaling pathways respectively. **d** High-ranked upregulated genes in ATM signaling pathway and downregulated genes in TGF-β signaling pathway were validated by qRT-PCR (*, *P* < 0.05; **, *P* < 0.001). **e** Western blot analysis indicated that ATM and histone H2AX were activated, while phosphorylation of Smad2 and Smad3 was reduced due to FGF18 knockdown. **f** Immunofluorescent staining validated that γH2AX was significantly increased in cells with FGF18 knockdown

To reveal the key signaling pathways related to FGF18 signaling in GC, expression microarray experiments were applied in siFGF18-transfected cells and negative controls. Through gene ontology (GO) analysis, genes that were downregulated in both siFGF18 transfected cells were screened as candidates for validation (Fig. 3a). This batch of genes were mainly enriched in four signaling pathways (Fig. 3b and Supplementary Table S1). In detail, ATM signaling and HDAC class III signaling were activated, while TGF-β and ATR pathways were suppressed (Fig. 3c). Genes enriched in ATM and TGF-β cascades were further validated by qRT-PCR. As shown in ATM signaling, *ATM*, *CTBP1*, and *TP53BP1* were upregulated in both siFGF18 transfected GC cell lines. In TGF-β signaling pathway, *TGFBR1*, *TGFBR2*, *ARRB2*, and *BAMB1* were downregulated in siFGF18 transfectants (*, *P* < 0.05; **, *P* < 0.001; Fig. 3d). Since ATM signaling pathway plays an imperative role in DNA repair and cell cycle regulation, the activation of ATM signaling pathway was subsequently validated. After transfecting siFGF18, the phosphorylated ATM and downstream factor γH2AX were activated in AGS and MKN28 cells. Meanwhile, as the functional components in TGF-β signaling pathway, phosphorylated Smad2 and phosphorylated Smad3 were inactivated (Fig. 3e). The immunofluorescence staining further confirmed the enhanced DNA damage caused by siFGF18 treatment (Fig. 3f).

### Autocrine secretion of FGF18 promotes tumor growth in GC

**Fig. 4:**
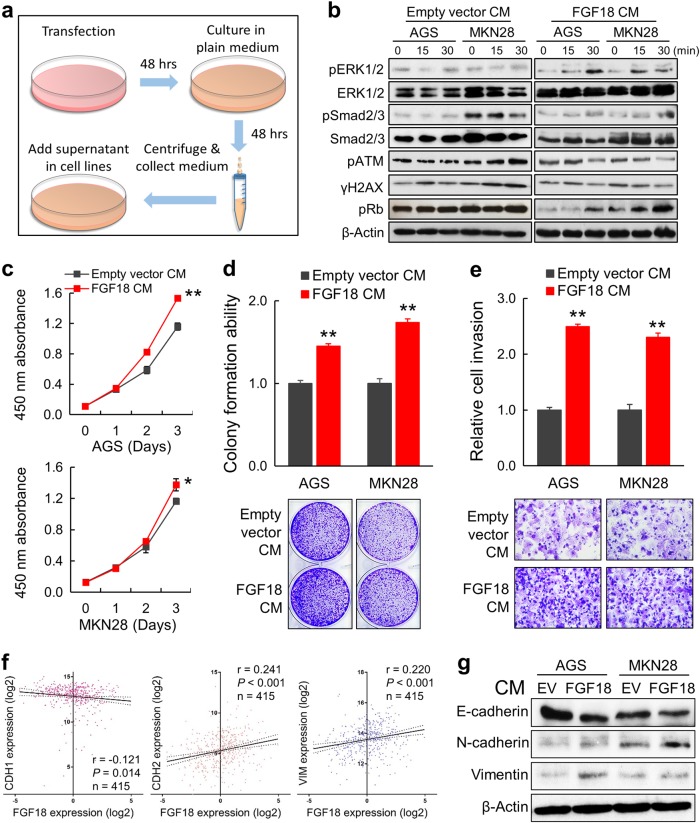
FGF18-conditioned medium (CM) enhances tumor growth of GC cells. **a** Schematic diagram for the CM preparation and cell treatment. **b** pERK1/2, pSMAD2/3, and pRb were activated by FGF18-CM, while ATM cascade was inactivated by FGF18-CM treatment. All changes were time-dependent. Cells under empty vector (EV)-CM treatment were applied as control. **c** Three-day cell proliferation assays indicated that FGF18-CM significantly increased proliferation of the GC cells (*, *P* < 0.05; **, *P* < 0.001). The mean and SDs obtained from six wells were plotted. **d** FGF18-CM enhanced the anchorage-dependent colony formation ability (**, *P* < 0.001). Assays were conducted in triplicate independently. Error bars represent SDs. **e** Treating with FGF18-CM accelerated cell invasion ability (**, *P* < 0.001). SDs were achieved from the cell number in each random vision field. **f** Correlation between FGF18 and related EMT markers based on the TCGA data. **g** Immunoblotting of EMT markers in the cells treated with CM for 48 h (empty vector and FGF18)

To mimic the autocrine secretion of FGF18 by GC cells, conditioned medium (CM) derived from cells with FGF18 overexpression was centrifuged and added in GC cells (Fig. 4a). Medium collected from cells transfected with empty vector (EV) was used as a control. Notably, phosphorylation of ERK1/2 and Smad2/3 were both elevated time dependently, while the pATM and γH2AX were decreased after FGF18-CM stimulation. Increased level of a cell-cycle regulatory molecule pRb was also observed by FGF18-CM (Fig. 4b). As to the functional effect, treating cells with FGF18-CM significantly accelerated proliferation rate, which was demonstrated by cell proliferation assay (*, *P* < 0.05; **, *P* < 0.001; Fig. 4c) and monolayer colony formation (*P* < 0.001; Fig. 4d) assays. More importantly, the cell invasion ability was enhanced after FGF18-CM treatment (*P* < 0.001; Fig. 4e). However, there were no similar changes of these related proteins in the cells treated with EV-CM. By analyzing TCGA cohort, the mRNA expression of FGF18 was negatively associated with CDH1 (E-cadherin), but positively correlated with CDH2 (N-cadherin) and VIM (Vimentin) (Fig. 4f). As well, the addition of FGF18-CM resulted in the decreased protein level of E-cadherin but increased exprsssion of N-cadherin and Vimentin, suggesting FGF18 promoted epithelial–mesenchymal transition (EMT) in GC cells (Fig. 4g). Together, these findings revealed an oncogenic role of autocrine FGF18 secretion in gastric tumorigenesis.

### FGF18 is negatively regulated by miR-590-5p

**Fig. 5:**
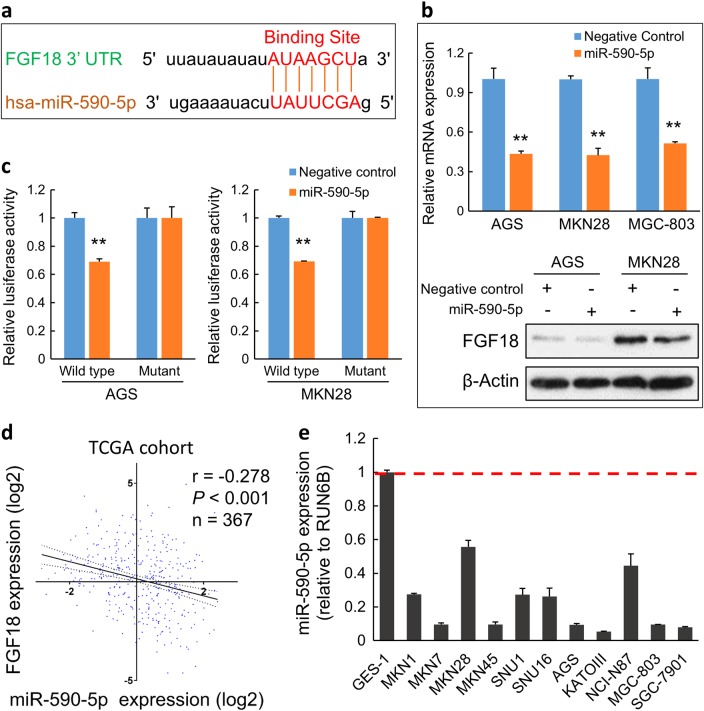
FGF18 is directly regulated by miR-590-5p in GC. **a** The putative binding site of miR-590-5p was located in the FGF18 3′ UTR according to miRNA database miRDB. **b** Both mRNA and protein expression of FGF18 were decreased after miR-590-5p overexpression in AGS and MKN28 cells (**, *P* < 0.001). **c** miR-590-5p suppressed the luciferase activity in the constructs containing the wild-type binding site in 3′ UTR of FGF18 (**, *P* < 0.001), but without effect in the constructs containing a corresponding mutated binding site. **d** The expression association between FGF18 mRNA and miR-590-5p in TCGA cohort. **e** The expression pattern of miR-590-5p in 11 GC cell lines and an immortalized gastric epithelium cell line GES-1. All GC cell lines showed a uniform decrease of miR-590-5p. Dash line indicated the normalized expression of miR-590-5p in GES-1

As the FGF18 upregulation in GC cells was not positively correlated with its copy number gain even amplification, other regulatory mechanisms such as microRNA (miRNA) regulation were considered. From the miRNA database miRDB, the 3′ untranslated region (UTR) of FGF18 was found to have a putative binding site for miR-590-5p (Fig. 5a) [14, 15]. To assess the regulation effect of miR-590-5p on FGF18, miR-590-5p precursor and negative control were transfected into AGS, MKN28, and MGC-803, respectively. Both mRNA and protein levels of FGF18 were decreased after ectopic expression of miR-590-5p (*P* < 0.001; Fig. 5b). Luciferase activity in cells containing the wild-type binding site of FGF18 was significantly inhibited by miR-590-5p, but the suppression effect of miR-590-5p was not detected in cells transfected with the construct containing the mutant binding site (Fig. 5c). The analysis of TCGA cohort also indicated that the mRNA level of FGF18 was negatively associated with miR-590-5p expression (*r* = −0.278, *P* < 0.001, *n* = 367; Fig. 5d). In all the eleven GC cell lines, there was a uniformly reduced expression of miR-590-5p compared with GES-1 (Fig. 5e). These results demonstrated that the upregulation of FGF18 in GC is partly due to the silence of miR-590-5p.

### miR-590-5p plays a tumor suppressor role in GC

**Fig. 6:**
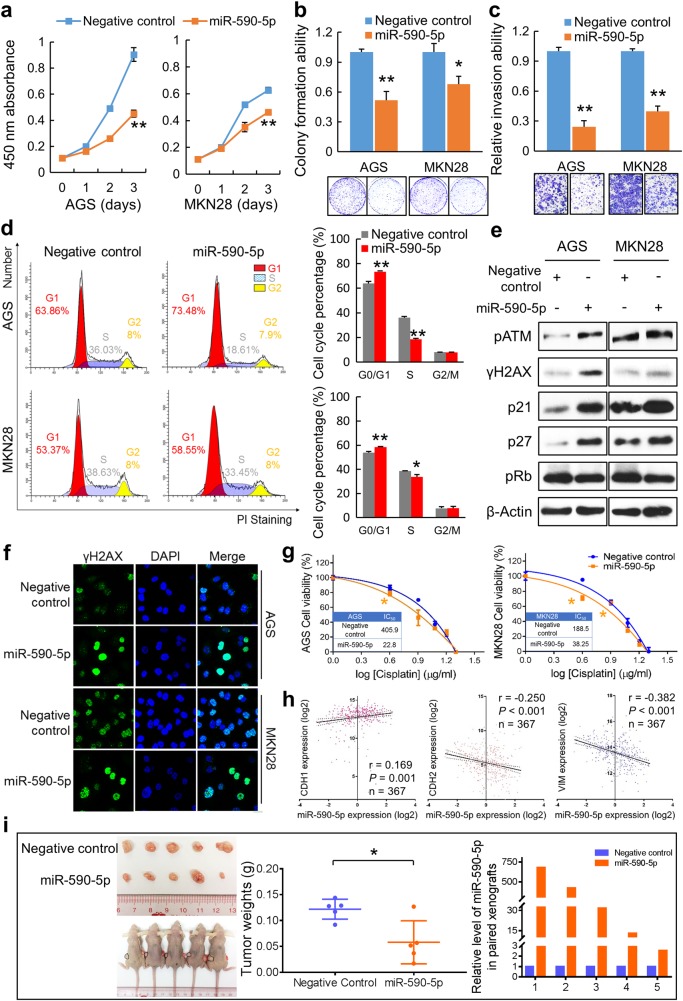
miR-590-5p functions as a tumor suppressor in GC. **a** Ectopic transfection of miR-590-5p significantly suppressed proliferation in GC cell lines (**, *P* *<* 0.001). The mean and SDs obtained from six wells were plotted. **b** miR-590-5p transfection significantly inhibited anchorage-dependent colony formation ability (**, *P* < 0.001). Experiments were performed in triplicate. Error bars represent SDs. **c** Cell invasion ability was suppressed by miR-590-5p significantly (*, *P* < 0.05; **, *P* < 0.001). SDs were achieved from visions randomly selected. **d** Cell cycle distribution was examined by flow cytometry, which suggested G1 phase arrest in miR-590-5p-transfected cells. Experiments were conducted in triplicate. Statistical analysis of cell cycle percentages were presented by histograms (*, *P* < 0.05; **, *P* < 0.001). **e** Western blot analysis demonstrated the increased level of p21, p27, and reduction of pRb. Key factors in ATM signaling were activated in the miR-590-5p transfectants. **f** Immunofluorescence showed that γH2AX was significantly increased in GC cells with ectopic miR-590-5p expression. **g** Drug sensitivity was enhanced by miR-590-5p (*, *P* < 0.05). The cell viability was detected with different concentrations of Cisplatin. The mean and SDs were obtained from six wells. The largest mean was defined as 100% and the smallest mean defined as 0%. IC_50_ values were calculated and listed in tables. **h** The expression correlation of miR-590-5p and related EMT markers in TCGA dataset. **i** The xenograft formation ability with stable miR-590-5p abundance was significantly inhibited compared with the negative control (*, *P* < 0.05). Black circles indicate the negative controls and red circles show the xenografts derived from miR-590-5p-transfected cells

To investigate the functional role of miR-590-5p in GC, the miR-590-5p precursor was transfected in GC cells. Cell proliferative (cell proliferation assays: *P* < 0.001, Fig. 6a; monolayer colony formation assays: *, *P* < 0.05, **, *P* < 0.001, Fig. 6b) and invasive abilities (*P* < 0.001; Fig. 6c) were all inhibited in response to miR-590-5p transfection. Furthermore, cell cycle distribution was affected by miR-590-5p, which was suggested by apparent G0/G1 phase arrest (*, *P* < 0.05; **, *P* < 0.001; Fig. 6d). From the western blot analysis, cell cycle regulators such as pRb were decreased, but p21 and p27 were activated. The increased level of phosphorylated ATM and γH2AX indicated the activation of ATM signaling (Fig. 6e), which was further confirmed by immunofluorescent staining (Fig. 6f). Thus, ectopic overexpression of miR-590-5p suppressed tumor formation, promoted G1 phase cell cycle arrest, and induced DNA damage. The therapeutic implication of miR-590-5p in GC cells were evaluated. Based on the IC_50_ (Fig. 6g), cells were more sensitive to Cisplatin when transfected with miR-590-5p, indicating its potential value as a promising therapeutic strategy. On the contrary with FGF18, miR-590-5p expression had positive correlation with CDH1 but negative correlation with CDH2 and VIM in TCGA cohort (Fig. 6h), suggesting the inhibitory role of miR-590-5p on EMT. The animal experiments further confirmed the inhibitory effect of miR-590-5p in the xenograft formation (*, *P* < 0.05; Fig. 6i). Meanwhile, the miR-590-5p expression in the xenografts was examined and miR-590-5p retained a significantly high level in the xenografts compared with negative control (Fig. 6i).

### Re-overexpression of FGF18 partly abolished the tumor-suppressive effect of miR-590-5p

**Fig. 7:**
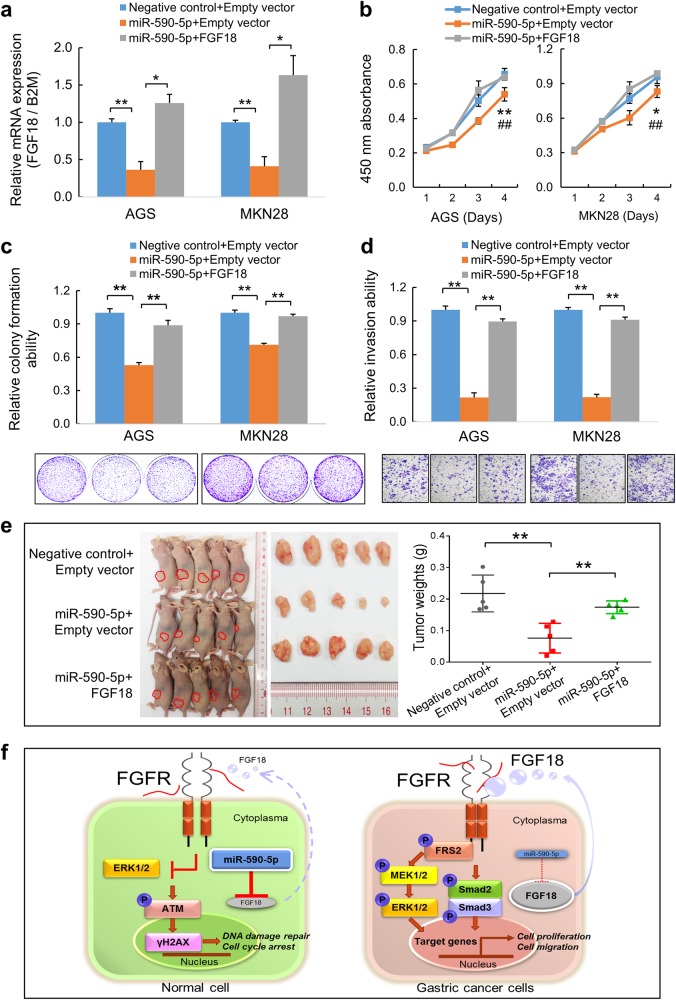
Re-overexpression diminished the suppressive effects of miR-590-5p in GC. **a** FGF18 mRNA was restored in FGF18 re-overexpressed AGS and MKN28 cells (*, *P* < 0.05; **, *P* < 0.001). **b** FGF18 re-overexpression promoted growth in the miR-590-5p treated cells (**, Negative control + Empty vector vs. miR-590-5p + Empty vector, *P* < 0.001; ##, miR-590-5p + Empty vector vs. miR-590-5p + FGF18, *P* < 0.001). **c** Monolayer colony formation ability of AGS and MKN28 cells, which were transfected with miR-590-5p, were rescued by FGF18 re-overexpression (**, *P* < 0.001). **d** The invasive ability was significantly raised in FGF18 re-overexpressed cells compared with miR-590-5p treated cells (**, *P* < 0.001). SDs were achieved from triplicate experiments. **e** FGF18 re-overexpression in MGC-803 cells formed bigger xenografts compared with miR-590-5p transfection group (**, *P* < 0.001). **f** Schematic figure summarized all the study. In normal gastric epithelium cells, miR-590-5p suppresses FGF18 expression and blocks FGF18 signaling. The ATM signaling is normally activated, which in turn induces DNA damage repair, cell cycle arrest and apoptosis. In GC cells, silenced miR-590-5p fails to inhibit FGF18 expression. Thus the activated FGF18 axis triggers MEK-ERK and Smad2/3 signalings through FGFR, which promotes downstream expression and causes aberrant proliferation and invasion

To confirm FGF18 is a real target of miR-590-5p, FGF18 was co-overexpressed with miR-590-5p in GC cells. The decreased mRNA level of FGF18 by miR-590-5p was restored (*, *P* < 0.05; **, *P* < 0.001; Fig. 7a). Moreover, the anti-tumor effect of miR-590-5p was partly rescued. Since miR-590-5p-inhibited cell proliferation ability in GC cells, re-overexpressing FGF18 in these cells reversed the cell proliferation rate to the level of negative control (*, *P* < 0.05; **, *P* < 0.001; ^##^, *P* < 0.001; Fig. 7b). As well, colony formation ability were significantly restored in miR-590-5p and FGF18 co-transfectants (**, *P* < 0.001; Fig. 7c). Similar results were observed in cell invasion assays (**, *P* < 0.001; Fig. 7d). In vivo, re-overexpression of FGF18 significantly accelerated the tumor formation compared with the miR-590-5p-treated group (Fig. 7e). Schematically, in normal gastric epithelial cells due to a normal expression of miR-590-5p, FGF18 expression was suppressed and the related downstream network was attenuated. The ATM signaling was thus activated in response to DNA damage repair and cell cycle progression was arrested. In gastric carcinogenesis, downregulation of miR-590-5p leads to an increase of FGF18, which further activates MEK-ERK signaling as well as Smad2 and Smad3, the key factors in TGF-β signaling pathway, to facilitate cell proliferation and migration (Fig. 7f).

## Discussion

In previous reports, the involvement of FGF18 has been addressed in multiple types of solid tumor, highlighting its oncogenic role in angiogenesis and cell growth. However, studies about the biological and prognostic role of FGF18 in GC remain uninvestigated. Here, we provide the first evidence that FGF18 is an oncogenic factor in GC and negatively regulated by tumor suppressor miRNA, miR-590-5p. FGF18 enrichment predicts poor survival in primary GC samples. FGF18 knockdown exerts tumor suppressive effect on GC cells both in vitro and in vivo. The activation of FGF18 is partly due to the downregulation of miR-590-5p in GC.

FGFs are produced by cancer cells or secreted by stromal compartment. Deregulation of FGF signaling has been found in both tumor cells and the cells in the tumorous environment [16, 17]. FGFs binds to their receptors and triggers dimerization and cross-phosphorylation of the kinase domains, subsequently, downstream effectors are activated [18]. When FGF signaling is activated, the phosphorylated FGFR substrate 2 (FRS2) recruits son of sevenless (SOS) and growth factor receptor-bound 2 (GFRB2), this complex further induces the activation of RAS-RAF-MEK-ERK signaling pathway which mediates cell proliferation, differentiation, and migration [17, 19]. A couple of studies provided evidences to demonstrate the role of FGF members in different cancer types via ERK-MAPK signaling pathway. FGF2 was indicated to regulate the FGFR-ERK signaling in esophageal squamous cell carcinoma to accelerate tumor growth [20]. In hepatocellular carcinoma, FGF8 subfamily members, including FGF8, FGF17 and FGF18, were upregulated to facilitate cell survival and angiogenesis via activating ERK [21]. The involvement of FGF7 and FGF9 secreted by cancer-associated fibroblasts were also demonstrated to promote migration and invasion of GC cells [22, 23]. However, apart from paracrine manner, the secretion of FGFs by cancer cells themselves also contributes to cancer cell growth [24]. The function of FGF ligands secreted by GC cells remains unknown. In our study, FGF18 was demonstrated to be a prominent ligand released by GC cells which functions as a potential oncogene to promote gastric carcinogenesis. On one hand, downregulated FGF18 led to cell growth inhibition and cell-cycle arrest. Herein, activation of ATM/γH2AX cascade and inactivation of TGF-β/SMAD signaling were revealed. It implied that DNA damage was caused by FGF18 knockdown, since the activation of ATM/γH2AX cascade was responsible for DNA double-strand break [25]. On the other hand, we also confirmed FGF18 derived CM significantly enhanced proliferation and invasion ability of GC cells. EMT properties were exhibited in the cells treated with FGF18-CM. TGF-β/SMAD signaling was proposed to play the mediated and crucial role in this process. It has been implicated that TGF-β/SMAD signaling accelerates cell migration, invasion, as well as EMT in the late stage of cancer [26]. One of the most prominent finding of FGFR in GC is the overexpression and amplification of FGFR2. It thus makes FGFR2 a significant prognostic and therapeutic target of GC [27–30]. Our results comprehensively delineated the detailed mechanisms of FGF18-FGFR2 signaling in gastric tumorigenesis.

In our work, we found FGF18 upregulation was not due to it copy number gain and amplification in GC. Therefore, we proposed miRNA regulation might be an important regulatory mechanism for the activation of FGF18. Accumulative evidence pointed out that miRNAs were potent molecular biomarkers for early cancer diagnosis and prognosis [31–33]. miR-590 has paradoxical roles in different cancer development [34–37]. In particular, it has been considered as an oncomiR and well characterized in colorectal cancer (CRC). As previously indicated, miR-590-5p was involved in the NF90/VEGFA signaling axis and inhibited angiogenesis and metastasis of CRC [38]. Another study in CRC also showed that the dysregulated DICER1-miR-590-5p axis led to the increased expression of YAP1 and promoted tumorigenesis [39]. In GC, the biological functions and underlying mechanisms of miR-590-5p are barely known. For the first time, our study delineates that miR-590-5p functions as a tumor suppressor by targeting FGF18, which broadens our knowledge regarding the target pool of this miRNA in gastric tumorigenesis.

In conclusion, our study not only identified a novel oncogenic FGF member, FGF18, in promoting GC, but also deciphered the regulatory mechanism between FGF18 signaling and miR-590-5p, which might imply a constructive therapeutic intervention in GC.

## Materials and methods

### GC cell lines

Human GC cell lines and commercial gastric-derived RNA sample applied in the study have been described [40, 41].

### In vitro functional studies

siRNAs and miRNAs transfection was conducted accordingly [42]. FGF18 siRNAs (SI03157896, SI03234679, SI03650318) were commercially available from Qiagen (Valencia, CA), while miRNA precursor miR-590-5p (PM11386, AM17110) were from Life Technologies.

Conditioned medium was prepared in 293 cells with FGF18 overexpression. Briefly, FGF18 plasmid and the corresponding empty vector were transfected into 293 cells for 48 h respectively. Then cells were maintained in RPMI 1640 plain medium for another 48 h. Conditioned medium was collected and centrifuged to remove the cell debris. Functional studies include cell proliferation, monolayer colony formation, cell invasion, and flow cytometry analysis for cell cycle distribution. Protocols were based on a previous study [43]. All experiments were conducted in triplicate.

### RNA isolation and quantitative real-time polymerase chain reaction (qRT-PCR)

The procedures of total RNA extraction and qRT-PCR were mentioned previously [44]. Primer sequences of genes in this study were listed in Supplementary Table S2.

### Western blot analysis

Primary antibodies were mainly from Cell Signaling (Danvers, MA), including Phospho-MEK1/2 (1:1000, #9121), Phospho-p44/42 MAPK (1:1000, #9106), Phospho-Rb (Ser807/811) (1:1000, #9308), p21 (1:1000, #2946), p27 (1:1000, #2552), Cyclin D1 (1:1000, #2978), CDK4 (1:1000, #12790), CDK6 (1:1000, #3136), γH2AX (1:1000, #9718), Histone H2AX (1:1000, #7631), pSmad2/3 (1:1000, #8828), Smad2/3 (1:1000, #3102), and GAPDH (1:1000, #2118). Some other antibodies are pATM (1:1000, ab81292), ATM (1:1000, ab32420), E-cadherin (1:500, AAS89512C, Antibody Verify), N-cadherin (1:1000, 33–3900, ZYMED), Vimentin (1:500, AAS26482C, Antibody Verify). And the rest antibodies have been reported earlier [41].

### Immunofluorescence staining

Primary antibodies included γH2AX (1:1000, #9718) and Histone H2AX (1:1000, #7631, Cell Signaling). The performance was based on the previous description [45].

### Luciferase assays

Sequences of the oligonucleotides were also listed in Supplemental Table S2. Luciferase activity was measured as before [46].

### Cisplatin sensitivity

The detailed method has been indicated in our previous study [42].

### Xenograft formation assays

The protocol of in vivo xenograft formation assay has been detailed described in our previous study [46]. All animal experiments were performed under the approval of Department of Health, Hong Kong and CUHK Animal Ethics Committee.

### Statistical analysis

Log transformation was needed for parametric tests when it is necessary. Corresponding statistical methods for each comparison and correlation was applied according to the previous study [46]. All the statistical results were analyzed upon SPSS software (version 22.0; SPSS Inc., Chicago, IL, USA; two-tailed, *P* *<* 0.05 was considered as statistically significant; two-tailed, *P* *<* 0.001, highly statistically significant).

## Electronic supplementary material

Supplementary Table S1

Supplementary Table S2

## References

[1] Jemal A, Bray F, Center MM, Ferlay J, Ward E, Forman D (2011). Global cancer statistics. CA Cancer J Clin.

[2] McLean MH, El-Omar EM (2014). Genetics of gastric cancer. Nat Rev Gastroenterol Hepatol.

[3] Network CGAR. (2014). Comprehensive molecular characterization of gastric adenocarcinoma. Nature.

[4] Wiedemann M, Trueb B (2000). Characterization of a novel protein (FGFRL1) from human cartilage related to FGF receptors. Genomics.

[5] Belov AA, Mohammadi M (2013). Molecular mechanisms of fibroblast growth factor signaling in physiology and pathology. Cold Spring Harb Perspect Biol.

[6] Touat M, Ileana E, Postel-Vinay S, Andre F, Soria JC (2015). Targeting FGFR signaling in cancer. Clin Cancer Res.

[7] Ohbayashi N, Shibayama M, Kurotaki Y, Imanishi M, Fujimori T, Itoh N (2002). FGF18 is required for normal cell proliferation and differentiation during osteogenesis and chondrogenesis. Genes Dev.

[8] Shimokawa T, Furukawa Y, Sakai M, Li M, Miwa N, Lin YM (2003). Involvement of the FGF18 gene in colorectal carcinogenesis, as a novel downstream target of the beta-catenin/T-cell factor complex. Cancer Res.

[9] Davidson D, Blanc A, Filion D, Wang H, Plut P, Pfeffer G (2005). Fibroblast growth factor (FGF) 18 signals through FGF receptor 3 to promote chondrogenesis. J Biol Chem.

[10] Wei W, Mok SC, Oliva E, Kim SH, Mohapatra G, Birrer MJ (2013). FGF18 as a prognostic and therapeutic biomarker in ovarian cancer. J Clin Invest.

[11] Katoh Y, Katoh M (2006). FGF signaling inhibitor, SPRY4, is evolutionarily conserved target of WNT signaling pathway in progenitor cells. Int J Mol Med.

[12] Mootha VK, Lindgren CM, Eriksson KF, Subramanian A, Sihag S, Lehar J (2003). PGC-1alpha-responsive genes involved in oxidative phosphorylation are coordinately downregulated in human diabetes. Nat Genet.

[13] Subramanian A, Tamayo P, Mootha VK, Mukherjee S, Ebert BL, Gillette MA (2005). Gene set enrichment analysis: a knowledge-based approach for interpreting genome-wide expression profiles. Proc Natl Acad Sci USA.

[14] Wang X (2008). miRDB: a microRNA target prediction and functional annotation database with a wiki interface. RNA.

[15] Wong N, Wang X (2015). miRDB: an online resource for microRNA target prediction and functional annotations. Nucleic Acids Res.

[16] Takahashi JA, Fukumoto M, Igarashi K, Oda Y, Kikuchi H, Hatanaka M (1992). Correlation of basic fibroblast growth factor expression levels with the degree of malignancy and vascularity in human gliomas. J Neurosurg.

[17] Itoh N, Ornitz DM (2011). Fibroblast growth factors: from molecular evolution to roles in development, metabolism and disease. J Biochem.

[18] Beenken A, Mohammadi M (2009). The FGF family: biology, pathophysiology and therapy. Nat Rev Drug Discov.

[19] Gotoh N (2008). Regulation of growth factor signaling by FRS2 family docking/scaffold adaptor proteins. Cancer Sci.

[20] Maehara O, Suda G, Natsuizaka M, Ohnishi S, Komatsu Y, Sato F (2017). Fibroblast growth factor-2-mediated FGFR/Erk signaling supports maintenance of cancer stem-like cells in esophageal squamous cell carcinoma. Carcinogenesis.

[21] Gauglhofer C, Sagmeister S, Schrottmaier W, Fischer C, Rodgarkia-Dara C, Mohr T (2011). Up-regulation of the fibroblast growth factor 8 subfamily in human hepatocellular carcinoma for cell survival and neoangiogenesis. Hepatology.

[22] Huang T, Wang L, Liu D, Li P, Xiong H, Zhuang L (2017). FGF7/FGFR2 signal promotes invasion and migration in human gastric cancer through upregulation of thrombospondin-1. Int J Oncol.

[23] Sun C, Fukui H, Hara K, Zhang X, Kitayama Y, Eda H (2015). FGF9 from cancer-associated fibroblasts is a possible mediator of invasion and anti-apoptosis of gastric cancer cells. BMC Cancer.

[24] Marek L, Ware KE, Fritzsche A, Hercule P, Helton WR, Smith JE (2009). Fibroblast growth factor (FGF) and FGF receptor-mediated autocrine signaling in non-small-cell lung cancer cells. Mol Pharmacol.

[25] Marechal A, Zou L (2013). DNA damage sensing by the ATM and ATR kinases. Cold Spring Harb Perspect Biol.

[26] Katz LH, Li Y, Chen JS, Munoz NM, Majumdar A, Chen J (2013). Targeting TGF-beta signaling in cancer. Expert Opin Ther Targets.

[27] Xie L, Su X, Zhang L, Yin X, Tang L, Zhang X (2013). FGFR2 gene amplification in gastric cancer predicts sensitivity to the selective FGFR inhibitor AZD4547. Clin Cancer Res.

[28] Matsumoto K, Arao T, Hamaguchi T, Shimada Y, Kato K, Oda I (2012). FGFR2 gene amplification and clinicopathological features in gastric cancer. Br J Cancer.

[29] Shoji H, Yamada Y, Okita N, Takashima A, Honma Y, Iwasa S (2015). Amplification of FGFR2 gene in patients with advanced gastric cancer receiving chemotherapy: prevalence and prognostic significance. Anticancer Res.

[30] Ahn S, Lee J, Hong M, Kim ST, Park SH, Choi MG (2016). FGFR2 in gastric cancer: protein overexpression predicts gene amplification and high H-index predicts poor survival. Mod Pathol.

[31] Chen G, Tang Y, Wu JH, Liu FH (2014). Role of microRNAs in diagnosis and treatment of the pathogenesis of gastric cancer. Int J Clin Exp Med.

[32] Wang QX, Zhu YQ, Zhang H, Xiao J (2015). Altered MiRNA expression in gastric cancer: a systematic review and meta-analysis. Cell Physiol Biochem.

[33] Ishiguro H, Kimura M, Takeyama H (2014). Role of microRNAs in gastric cancer. World J Gastroenterol.

[34] Wang FF, Wang S, Xue WH, Cheng JL (2016). microRNA-590 suppresses the tumorigenesis and invasiveness of non-small cell lung cancer cells by targeting ADAM9. Mol Cell Biochem.

[35] Zhou L, Zhao LC, Jiang N, Wang XL, Zhou XN, Luo XL (2017). MicroRNA miR-590-5p inhibits breast cancer cell stemness and metastasis by targeting SOX2. Eur Rev Med Pharmacol Sci.

[36] Liu Y, Wang F, Xu P (2017). miR-590 accelerates lung adenocarcinoma migration and invasion through directly suppressing functional target OLFM4. Biomed Pharmacother.

[37] Chu Y, Ouyang Y, Wang F, Zheng A, Bai L, Han L (2014). MicroRNA-590 promotes cervical cancer cell growth and invasion by targeting CHL1. J Cell Biochem.

[38] Zhou Q, Zhu Y, Wei X, Zhou J, Chang L, Sui H (2016). MiR-590-5p inhibits colorectal cancer angiogenesis and metastasis by regulating nuclear factor 90/vascular endothelial growth factor A axis. Cell Death Dis.

[39] Ou C, Sun Z, Li X, Ren W, Qin Z, Zhang X (2017). MiR-590-5p, a density-sensitive microRNA, inhibits tumorigenesis by targeting YAP1 in colorectal cancer. Cancer Lett.

[40] Zhou Y, Huang T, Zhang J, Wong CC, Zhang B, Dong Y (2017). TEAD1/4 exerts oncogenic role and is negatively regulated by miR-4269 in gastric tumorigenesis. Oncogene.

[41] Kang W, Huang T, Zhou Y, Zhang J, Lung RWM, Tong JHM (2018). miR-375 is involved in Hippo pathway by targeting YAP1/TEAD4-CTGF axis in gastric carcinogenesis. Cell Death Dis.

[42] Zhang J, Zhou Y, Huang T, Wu F, Liu L, Kwan JSH (2018). PIEZO1 functions as a potential oncogene by promoting cell proliferation and migration in gastric carcinogenesis. Mol Carcinog.

[43] Kang W, Tong JH, Lung RW, Dong Y, Zhao J, Liang Q (2015). Targeting of YAP1 by microRNA-15a and microRNA-16-1 exerts tumor suppressor function in gastric adenocarcinoma. Mol Cancer.

[44] Zhou Y, Huang T, Siu HL, Wong CC, Dong Y, Wu F (2017). IGF2BP3 functions as a potential oncogene and is a crucial target of miR-34a in gastric carcinogenesis. Mol Cancer.

[45] Huang T, Zhou Y, Zhang J, Wong CC, Li W, Kwan JSH (2018). SRGAP1, a crucial target of miR-340 and miR-124, functions as a potential oncogene in gastrictumorigenesis. Oncogene.

[46] Huang T, Kang W, Zhang B, Wu F, Dong Y, Tong JH (2016). miR-508-3p concordantly silences NFKB1 and RELA to inactivate canonical NF-kappaB signaling in gastric carcinogenesis. Mol Cancer.

